# Two new species of the bamboo-feeding planthopper genus *Purohita* Distant from China (Hemiptera, Fulgoromorpha, Delphacidae)

**DOI:** 10.3897/zookeys.855.31561

**Published:** 2019-06-13

**Authors:** Hong-Xing Li, Lin Yang, Xiang-Sheng Chen

**Affiliations:** 1 Institute of Entomology, Guizhou University, Guiyang, Guizhou University Guiyang China; 2 Guizhou, 550025, China Guizhou University Guiyang China; 3 The Provincial Special Key Laboratory for Development and Utilization of Insect Resources, Guizhou University, Guiyang, Guizhou, 550025, China Guizhou University Guiyang China

**Keywords:** Bamboo planthopper, Fulgoroidea, morphology, taxonomy

## Abstract

Two new species of the bamboo-feeding genus *Purohita* Distant, 1906, *P.castaneus***sp. nov.** and *P.circumcincta***sp. nov.**, are described and illustrated from southwest China (Yunnan), giving the genus thirteen species in total. A key is provided to distinguish eight Chinese species in the genus.

## Introduction

Distant (1906) established the bamboo-feeding planthopper genus *Purohita* with the type species *P.cervina* Distant, 1906 from Ceylon. This genus belongs to the tribe Tropidocephalini of subfamily Delphacinae (Hemiptera, Fulgoromorpha, Delphacidae) and is easily recognized from other members in this tribe by the very large antennae, first segment flattened, rectangular, longer than the second segment. Yang and Yang (1986) revised the species of *Purohita* and divided the five known species in Taiwan, China into three subgenera. The first to fifth instars of *P.taiwanensis* Muir, 1914, are also described by Yang and Yang (1986). So far, 11 species of *Purohita* are described, including from China (seven species: *P.fuscovenosa* Muir, *P.maculata* Muir, *P.nigripes* Muir, *P.picea* Yang & Yang, *P.sinica* Huang & Ding, *P.taiwanensis* Muir and *P.theognis* Fennah) (Muir 1913, 1916; Huang et al. 1979; Yang and Yang 1986; Ding 2006; Hayashi and Fujinuma 2016), India (two species: *P.arundinacea* Distant and *P.punjabensis* Sharma & Singh) (Distant 1907; Sharma and Singh 1982), Ceylon (one species: *P.cervina* Distant) (Distant 1906), Japan (two species: *P.cervina* Distant and *P.taiwanensis* Muir) (Hayashi and Fujinuma 2016), Philippine (one species: *P.nigripes* Muir) (Muir 1916; Ding 2006), Pakistan (one species: *P.qadrii* Jabbar-Khan & Jabbar-Khan) (Jabbar-Khan and Jabbar-Khan 1985) and Vietnam (one species: *P.theognis* Fennah) (Fennah 1978; Ding 2006).

Species of *Purohita* with reported plant associations feed on bamboo (Distant 1906; Muir 1913; Huang et al. 1979; Yang and Yang 1986; Ding 2006; this paper). These members were always collected on several genera of bamboos including *Sinocalamus*, *Bambusa*, *Pheioblastus*, *Phyllostachys* and *Dendrocalamus* (Huang et al. 1979; Yang and Yang 1986; Ding 2006). *P.taiwanensis* Muir is of economic significance since the species has large populations in the bamboo fields and is widely distributed in southern China.

Herein, two new species: *Purohitacastaneus* sp. nov. and *P.circumcincta* sp. nov. are described and illustrated from Yunnan province, China. A key to species of *Purohita* from China is provided.

## Materials and methods

The morphological terminology and measurements follow Yang and Yang (1986). Body length was measured from apex of vertex to tip of tegmina. Dry male specimens were used for the description and illustration. External morphology was observed under a stereoscopic microscope and characters were measured with an ocular micrometer. Color pictures for adult habitus were obtained by the KEYENCE VHX-1000 system. The genital segments of the examined specimens were macerated in 10% KOH and drawn from preparations in glycerin jelly using a Leica MZ 12.5 stereomicroscope. Illustrations were scanned with a Canon CanoScan LiDE 200 and imported into Adobe Photoshop 6.0 for labeling and plate composition.

The type specimens of the new species are deposited in the Institute of Entomology, Guizhou University, Guiyang, China (**IEGU**).

## Taxonomy

### Purohita

Taxon classificationAnimaliaHemipteraDelphacidae

Distant, 1906

Purohita Distant, 1906: 470; Ishihara 1949: 86; Tian 1983: 43; Yang and Yang 1986: 64; Ding 2006: 201.

#### Type species.

*Purohitacervina* Distant, 1906, by original designation.

#### Diagnosis.

Head including eyes narrower than pronotum. Vertex narrow, quadrate, slightly extending in front of eyes; lateral carinae strongly ridged, foliate, prominent anteriorly, submedian carinae transverse, median carina obsolete. Frons in middle line longer than wide at widest part about 1.5–2.3: 1, lateral margins divergent apically, median carina forked near base. Postclypeus tricarinate. Antennae very large, first segment flattened, rectangular, longer in middle line than widest part about 2.9–3.3: 1, with central ridge distinct, surface on each side of central ridge obliquely reclined, second segment much shorter than the first about 1: 1.4–2.5. Eyes in dorsal view with lateral side emarginate medially distinctly. Ocelli distinct. Pronotum short, scarcely longer than vertex, tricarinate, lateral carinae attaining hind margin. Mesonotum longer than vertex and pronotum together, tricarinate. Spinal formula of hind leg 5-6-4 or 5-7-4. Wings with M and Cu_1_ fused except very short portion at base.

Anal segment large, broad, dorsum flattened and lateroapical angles without process. Pygofer slightly compressed laterally, medioventral processes present or absent. Aedeagus with phallus relative long, phallobase process arising from base, directed ventrad, blunt oval or forked at apex. Diaphragm and lateral areas membranous. Without sclerotized margin of opening of genital styles. Seventh abdominal sternite of female present or absent, genital styles narrow and slender, simple.

#### Plant associations.

Bamboo.

#### Distribution.

Oriental region.

##### Key to species (males) of *Purohita* from China (modified from Ding 2006)

**Table:** 

1	Tegmina with transverse veins (nodal line) bordered with brown stripe (see Ding 2006: fig. 104H)	*** P. theognis ***
–	Tegmina with transverse veins not bordered with brown stripe	**2**
2	Pygofer with distinct medioventral processes	**3**
–	Pygofer without medioventral process	**7**
3	Pygofer with medioventral processes protruding in front of margin deeply incised at apex; in posterior view genital styles with inner margin basal half extending quadrate	**4**
–	Pygofer with medioventral processes not protruding in front of margin, outer pair distinctly higher than median ones, median portion deeply cleft; in posterior view genital styles slender	**6**
4	Medioventral process bifurcated, hook-like	**5**
–	Medioventral process flattened, with minute production on outer side (see Huang et al. 1979: fig. 24)	*** P. sinica ***
5	Medioventral processes with apices directed dorsolaterally, each side with a process enlarging at apex, apical margin truncate (see Ding 2006: fig. 101C)	*** P. taiwanensis ***
–	Medioventral processes with apices directed dorsomedially, each side with a large triangular process (Fig. 7)	***P.castaneus* sp. nov.**
6	Medioventral processes with median ones each with two processes at apex (see Yang and Yang 1986: fig. 42E, H)	*** P. picea ***
–	Medioventral processes with median ones each with single process at apex (see Yang and Yang 1986: fig. 41B, D)	*** P. nigripes ***
7	Pygofer with medioventral margin V-like; anal segment with length longer than width more 1.6: 1 (Fig. 19)	***P.circumcincta* sp. nov.**
–	Pygofer with medioventral margin broadly U-shaped; anal segment with length longer than width about 1.1: 1 (see Yang and Yang 1986: fig. 40E)	*** P. maculata ***

### Purohita castaneus sp. nov.

Taxon classificationAnimaliaHemipteraDelphacidae

http://zoobank.org/47C580B3-61BE-4777-91AF-6163AFFBB699

Figs 1–12

#### Type material.

Holotype: ♂, **China**: Yunnan, Yingjiang County (24°44'N, 97°33'E), on bamboo, 17 August 2018, Hong-Xing Li; paratypes, 1♂, 3♀♀, same data as holotype, Hong-Xing Li and Qiang Luo.

#### Etymology.

Specific epithet derived from “*castaneus*”, referring to the brown color of the pronotum and mesonotum.

#### Measurements.

Body length (from apex of vertex to tip of tegmina): male 4.6–4.8 mm (n = 2); female 5.1–5.3 mm (n = 3); tegmen length: male 3.9–4.0 mm (n = 2); female 4.4–4.7 mm (n = 3).

#### Diagnosis.

The salient features of the new species include the following: pygofer with medioventral processes forming a bifurcate hook, apices directed dorsomedially, and each side with a large triangular process (Fig. 7).

#### Description.

**Figures 1–12.**
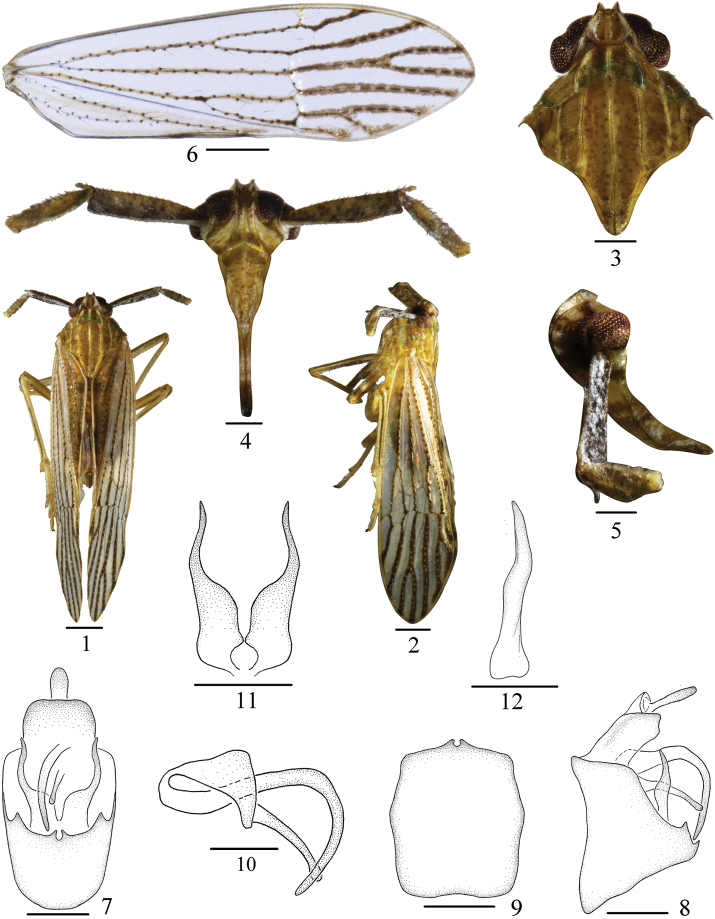
*Purohitacastaneus* sp. nov. **1** Male habitus, dorsal view **2** Same, lateral view **3** Head and thorax, dorsal view **4** Face **5** Frons and clypeus, lateral view **6** Forewing **7** Male genitalia, posterior view **8** Same, lateral view **9** Pygofer, ventral view **10** Aedeagus **11** Genital style, posterior view **12** Same, lateral view. Scale bars: 0.5 mm (**1, 2, 6**); 0.2 mm (**3–5, 7–12**).

*Coloration*. General color brown (Figs 1–6). Vertex yellowish brown. Frons with basal half brown speckled with milky white, thence milky white to apical quarter, the apical quarter yellowish brown. Genae milky white at basal two thirds and yellow at apical third. Clypeus yellowish brown. Rostrum black brown at apex. Antennae brown. Eyes reddish brown, ocelli red. Pronotum yellowish green, with lateral margins milky white. Mesonotum yellowish brown. Tegmina hyaline, veins with small hair-bearing granules, apical half bordered with black brown markings. Wings hyaline. Legs with longitudinal stripes, dark brown.

*Head and thorax*. Vertex (Figs 1, 3) shorter in middle line than wide at base (0.71: 1), width at apex narrower than at base (0.31: 1), anterior margin distinct sinuate, Y-shaped carina with stalk indistinct, with very short arms. Frons (Fig. 4) longer at midline than wide at widest part, about 1.73: 1, widest at apex. Base of postclypeus wider than apex of frons. Antennae (Fig. 4) with first segment rectangular, with central ridge, longer in middle line than widest part about 3.86: 1, longer than the second about 1.5: 1. Pronotum (Figs 1, 3) slightly shorter than vertex (0.73: 1). Mesonotum longer in middle line than vertex and pronotum together, about 2.08: 1, median carina reaching the end of scutellum, lateral carinae not attaining hind margin. Tegmen (Fig. 6) longer than widest portion about 3.58: 1. Spinal formula of hind leg 5-6-4.

*Male genitalia*. Anal segment (Figs 7, 8) at widest part narrower than pygofer, large, broad and flattened dorsoventrally. Anal style moderately long. Pygofer (Figs 7–9) with medioventral processes forming a bifurcate hook, apices directed dorsomedially, in lateral view, ventral margin of pygofer much longer than dorsal. Aedeagus (Fig. 10) with phallus slender, long, acute at apex, almost attached to ventral margin of pygofer, in lateral view, phallus turned in right angle at apical half. Phallobasal process rising from the base, long, blunt oval at apex. Genital styles (Figs 11, 12) large, curved inward at apical half, with basal half broad, then become of slender gradually, acute at apex.

#### Plant associations.

Bamboo.

#### Distribution.

Southwest China (Yunnan).

#### Remarks.

This species is similar to *P.taiwanensis* Muir, 1914 but differs from it by: (1) pygofer (Figs 7, 9) with medioventral processes forming a bifurcate hook, apices directed dorsomedially (medioventral processes with apices directed dorsolaterally in *P.taiwanensis*); (2) sides of medioventral processes of pygofer (Fig. 7) each with a large triangular process (sides of medioventral processes each with a process enlarging at apex, apical margin truncate in *P.taiwanensis*); (3) genital styles (Fig. 11) in posterior view basal half distinctly wider than apical half (genital styles in posterior view basal half slightly wider than apical half in *P.taiwanensis*).

This species is also similar to *P.sinica* Huang & Ding, 1979 but differs from it by: (1) pygofer (Figs 7, 9) with medioventral processes forming a bifurcate hook, without tooth on outer margin (medioventral process flattened, each with a small tooth on outer margin in *P.sinica*); (2) medioventral processes of pygofer (Figs 7, 9) with apices directed dorsomedially (medioventral processes with apices directed dorsolaterally in *P.sinica*); (3) sides of medioventral processes of pygofer (Fig. 7) each with a triangular process, which at a distance from the medioventral processes (each with a triangular process near the medioventral processes in *P.sinica*).

### Purohita circumcincta sp. nov.

Taxon classificationAnimaliaHemipteraDelphacidae

http://zoobank.org/809655E9-FFAB-402B-9768-95B4DEEB7F47

Figs 13–22

#### Type material.

Holotype: ♂, **China**: Yunnan, Yingjiang County (24°44'N, 97°33'E), on bamboo, 17 August 2018, Hong-Xing Li; paratypes, 2♂♂, 6♀♀, same data as holotype, Hong-Xing Li, Nian Gong, Liang-Jing Yang and Qiang Luo; paratypes, 1♂, 2♀♀, Yunnan, Yingjiang, on bamboo, 18 August 2015, Xiang-Sheng Chen and Lin Yang; paratypes, 1♂, 1♀, Yunnan, Ruili, on bamboo, 6 June 2011, Jian-Kun Long; paratypes, 1♂, 1♀, Yunnan, Mangshi, on bamboo, 8 June 2011, Yu-Jian Li.

#### Etymology.

The specific epithet refers to the pygofer without medioventral process.

#### Measurements.

Body length (from apex of vertex to tip of tegmina): male 5.2–5.7 mm (n = 6); female 6.3–6.8 mm (n = 10); tegmen length: male 4.4–4.9 mm (n = 6); female 5.3–5.9 mm (n = 10).

#### Diagnosis.

**Figures 13–22.**
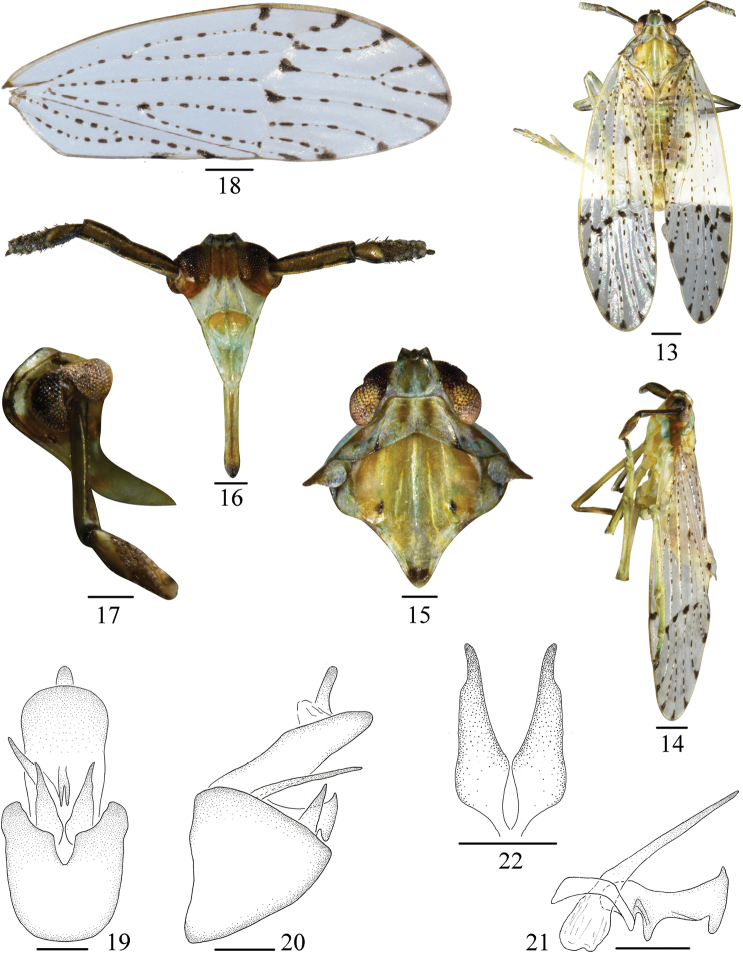
*Purohitacircumcincta* sp. nov. **13** Male habitus, dorsal view **14** Same, lateral view **15** Head and thorax, dorsal view **16** Face **17** Frons and clypeus, lateral view **18** Forewing **19** Male genitalia, posterior view **20** Same, lateral view **21** Aedeagus, lateral view **22** Genital style, posterior view. Scale bars: 0.5 mm (**13, 14, 18**); 0.2 mm (**15–17, 19–22**).

The salient features of the new species include the following: tegmina milky-hyaline, veins with black hair-bearing granules, many dark markings on veins (Figs 13, 18); pygofer with medioventral margin V-like (Fig. 19); aedeagus with phallus slender, acute at apex (Fig. 21).

#### Description.

*Coloration*. General color milky white to yellowish brown (Figs 13–18). Vertex yellowish green, submedian carinae brown. Frons yellow at basal half and milky white at apical half. Genae white. Postclypeus yellow at basal half and white at apical half. Anteclypeus milky white. Rostrum black brown at apex. Antennae yellowish brown. Eyes and ocelli reddish brown. Pronotum yellowish green, lateral margin milky white, with dark brown markings at apex of lateral carinae. Mesonotum yellowish green, with dark brown markings at near apex of lateral carinae and apex of scutellum. Tegmina milky white, hyaline, veins white with short dark brown stripes. Wings hyaline. Legs with longitudinal stripes, dark brown.

*Head and thorax*. Vertex (Figs 13, 15) quadrate, wider at base than length about 1.17: 1, width at apex narrower than at base (0.4: 1), anterior margin sinuate, carinae distinct, submedian carinae uniting at apex. Frons (Figs 16, 17) longer at midline than wide at widest part, about 1.19: 1, widest at apex, carinae distinct. Postclypeus as wide at base as frons at apex, median carina distinct. Antennae with first segment rectangular, with central ridge, longer in middle line than widest part about 3.67: 1, longer than the second about 1.48: 1. Pronotum (Figs 13, 15) shorter than vertex (0.72: 1). Mesonotum longer in middle line than vertex and pronotum together, about 1.57: 1, median carina reaching the end of scutellum, lateral carinae not attaining hind margin. Tegmen (Fig. 18) longer than widest portion about 3.7: 1. Spinal formula of hind leg 5-6-4.

*Male genitalia*. Anal segment (Figs 19, 20) at widest part narrower than pygofer, large, broad, flattened dorsoventrally and apex rounded. Anal style moderately long. Pygofer (Figs 19, 20) slightly compressed laterally, in posterior view with opening longer than wide, medioventral margin V-like. Aedeagus (Fig. 21) with phallus slender, long, with base broad, then become of slender gradually, acute at apex. Phallobasal forked at apex, in profile broad, apex with two finger-like processes and near base with a stout tooth-like process. Genital styles (Fig. 22) large, broad at base, tapering apically.

#### Plant associations.

Bamboo.

#### Distribution.

Southwest China (Yunnan).

#### Remarks.

This species is similar to *P.theognis* Fennah, 1978 but differs from it by: (1) tegmina (Fig. 18) with transverse veins milky white, without brown stripe border (tegmina with transverse veins bordered with brown stripe in *P.theognis*); (2) pygofer of male (Fig. 19) without medioventral process (pygofer with medioventral process in *P.theognis*); (3) genital styles (Fig. 22) with base broad, not twisted apically (genital styles slender, with apical quarter twisted in *P.theognis*).

This species is also similar to *P.maculata* Muir, 1916 but differs from it by: (1) pygofer of male (Fig. 19) with medioventral margin V-like (pygofer with medioventral margin broadly U-shaped in *P.maculata*); (2) anal segment of male (Fig. 19) with length longer than width, exceeding 1.6: 1 (anal segment with length longer than width about 1.1: 1 in *P.maculata*); (3) aedeagus (Fig. 21) with phallus slender, acute at apex (aedeagus with phallus with apex rounded in *P.maculata*).

## Discussion

In this paper, we describe two new species from China and provisionally place it in the genus *Purohita* based on the very large antennae, first segment flattened, rectangular, longer than the second segment. Ishihara, 1949: 16, noted “[*Purohitacervina* Distant, 1906] is the commonest species of the genus”, with the same note repeated in Yang and Yang (1986). Up till now we have no information about it in China. Therefore, *P.cervina* may be not widely distributed in China. Muir (1913) added one species, *P.fuscovenosa*, based on a female specimen from Macao, China. Unfortunately, we have not discovered the male specimen. The absence of males for comparison is regrettable.

## Supplementary Material

XML Treatment for Purohita

XML Treatment for Purohita castaneus

XML Treatment for Purohita circumcincta
